# Chemical Synthesis of TFF3 Reveals Novel Mechanistic Insights and a Gut-Stable Metabolite

**DOI:** 10.1021/acs.jmedchem.1c00767

**Published:** 2021-06-18

**Authors:** Nayara Braga Emidio, Rajeshwari Meli, Hue N. T. Tran, Hayeon Baik, Séverine Morisset-Lopez, Alysha G. Elliott, Mark A. T. Blaskovich, Sabrina Spiller, Annette G. Beck-Sickinger, Christina I. Schroeder, Markus Muttenthaler

**Affiliations:** † Institute for Molecular Bioscience, The University of Queensland, Brisbane, QLD 4072, Australia; ‡ Institute of Biological Chemistry, Faculty of Chemistry, University of Vienna, Vienna 1090, Austria; § Centre de Biophysique Moléculaire, CNRS, Unité Propre de Recherche 4301, Université d’Orléans, Orleans 45071, France; ∥ Institute of Biochemistry, Faculty of Life Sciences, Leipzig University, Leipzig 04103, Germany; ⊥ Center for Cancer Research, National Cancer Institute, National Institutes of Health, Frederick, Maryland 21702, United States

## Abstract

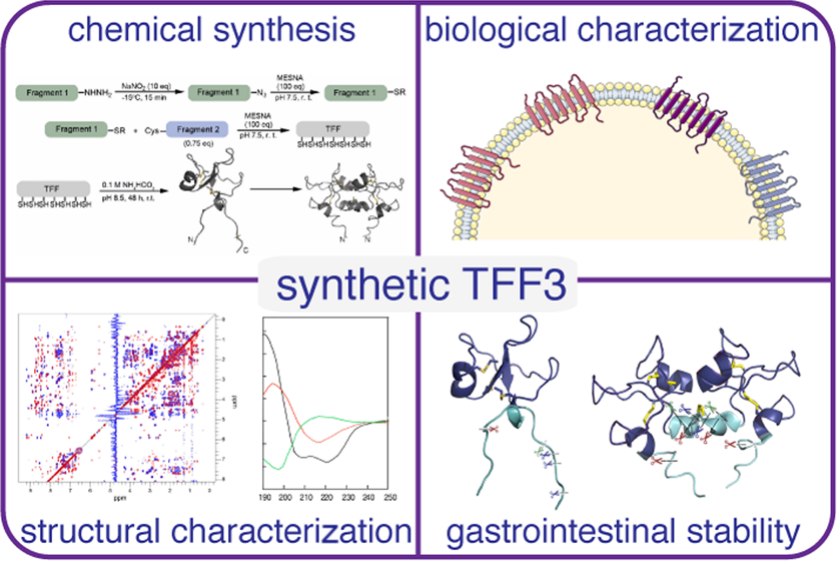

TFF3 regulates essential gastro- and neuroprotective functions, but its molecular mode of action remains poorly understood. Synthetic intractability and lack of reliable bioassays and validated receptors are bottlenecks for mechanistic and structure–activity relationship studies. Here, we report the chemical synthesis of TFF3 and its homodimer *via* native chemical ligation followed by oxidative folding. Correct folding was confirmed by NMR and circular dichroism, and TFF3 and its homodimer were not cytotoxic or hemolytic. TFF3, its homodimer, and the trefoil domain (TFF3_10-50_) were susceptible to gastrointestinal degradation, revealing a gut-stable metabolite (TFF3_7-54_; *t*_1/2_ > 24 h) that retained its trefoil structure and antiapoptotic bioactivity. We tried to validate the putative TFF3 receptors CXCR4 and LINGO2, but neither TFF3 nor its homodimer displayed any activity up to 10 μM. The discovery of a gut-stable bioactive metabolite and reliable synthetic accessibility to TFF3 and its analogues are cornerstones for future molecular probe development and structure–activity relationship studies.

## Introduction

The trefoil factor family (TFF) comprises three disulfide-rich peptides (TFF1, TFF2, TFF3) that are abundantly secreted in the gastrointestinal tract where they regulate gut homeostasis by promoting gut protection and repair.^1−5^ They are also expressed in mucosal tissues outside the gut, including in the respiratory tract, urinary tract, uterus, eyes, and salivary glands, where they have similar mucosal repair and protective functions. TFF peptides have also been observed in human breast milk and the brain,^1,6,7^ and have been implicated in cancer development.^1,8,9^

TFF3 is highly expressed in the gastrointestinal mucosa, particularly in the small intestine and colon, where it protects, maintains, and repairs the gastrointestinal tract.^4,10−17^ In the central nervous system (CNS), TFF3 is secreted by neurons and regulates physiological effects such as neuroinflammation^18^ and behavioral processes including learning and memory^19^ and depression.^20,21^ The anti-inflammatory effects of TFF3 on microglia cells (reduced expression and secretion of pro-inflammatory cytokines) and its capacity to mitigate ischemic cerebral injuries by reducing cell death *via* suppression of caspase-3 activity further support TFF3’s neuroprotective role in the CNS.^18,22^

TFF3 derives from a 94-residue-long precursor protein that comprises a 35-residue-long signal peptide followed by the 59-residue-long TFF3 sequence.^23^ The mature secreted and folded TFF3 peptide (TFF3_1-59_) contains a highly conserved trefoil domain (TFF3_10-50_) that also defines the other members of the TFF.^3,24^ The trefoil domain contains six conserved cysteine residues (CX_9-10_CX_9_CX_4_CCX_10_C motif) forming three intrachain disulfide bonds in the configuration Cys^I–V^, Cys^II-IV^, and Cys^III–VI^.^3,24^ This disulfide bond arrangement creates a compact three-loop structure resembling a trefoil shape, which is considered to be metabolically stable based on TFF3’s functional role in the gastrointestinal tract.^3,24,25^ Its gastrointestinal stability has, however, not been systematically investigated, and some reports indicate that TFF3 might not be that stable.^26−28^

TFF3 also has an additional *C*-terminal cysteine residue (Cys^VII^) located outside the trefoil domain, which enables the formation of covalent homo- or heterodimers (e.g., with the mucus-associated Fc fragment of IgG Fc binding protein, FCGBP).^29,30^ TFF3-FCGBP’s function remains unknown, but it is hypothesized to act as a TFF3 reservoir.^29^ Although the TFF3 homodimer is only present in relatively small quantities,^29^ it is more potent than its monomeric counterpart in promoting cell motility.^31,32^ Additionally, only the homodimer but not the monomer displays protective effects when luminally administrated in an experimental model of colitis.^32^

TFF3 can induce several biological effects (i.e., antiapoptotis,^33,34^ cell migration,^35−37^ and anti-inflammatory effects^38^), but its mode of action and target receptors have not been fully elucidated nor validated.^30^ TFF3 was recently described as a natural ligand of the leucine-rich repeat receptor and nogo-interacting protein 2 (LINGO2),^38^ with TFF3-LINGO2 interaction mediating intestinal wound healing and immunity through enhanced epidermal growth factor receptor (EGFR) signaling.^38^ Another putative receptor is the chemokine receptor type 4 (CXCR4), through which TFF3 might mediate cell migration *via* an ERK1/2-independent signaling pathway.^39^

TFF3 is considered a promising therapeutic lead, especially for disorders requiring epithelial protection, repair, or restitution, such as inflammatory bowel diseases (IBD) and nonsteroidal anti-inflammatory drug-induced gastritis.^30^ Its therapeutic potential is supported by promising preclinical^11,40−42^ and clinical studies.^30,43,44^ TFF3 is currently obtained either through recombinant production in *Escherichia coli*, *Saccharomyces cerevisiae*, or human embryonic kidney 293 (HEK-293) cells,^45−47^ or through purification from milk^48^ or meconium.^49^ These approaches are however not ideal for drug target discovery, where advanced probe development is required, nor for drug development efforts where the incorporation of unnatural amino acids, reporter tags, or conjugation handles at specific positions is often required. Chemical synthesis would allow for such regiospecific control and incorporation of unnatural amino acids, thereby considerably facilitating mechanistic studies and therapeutic development. We thus set out to establish a synthetic strategy to produce TFF3 and its homodimer reliably and in sufficient quantities to deliver new insights into their pharmacology, toxicity, and metabolic stability.

## Results

### Chemical Synthesis of TFF3, TFF3(C^57^Acm), and TFF3 Homodimer

**Figure 1:**
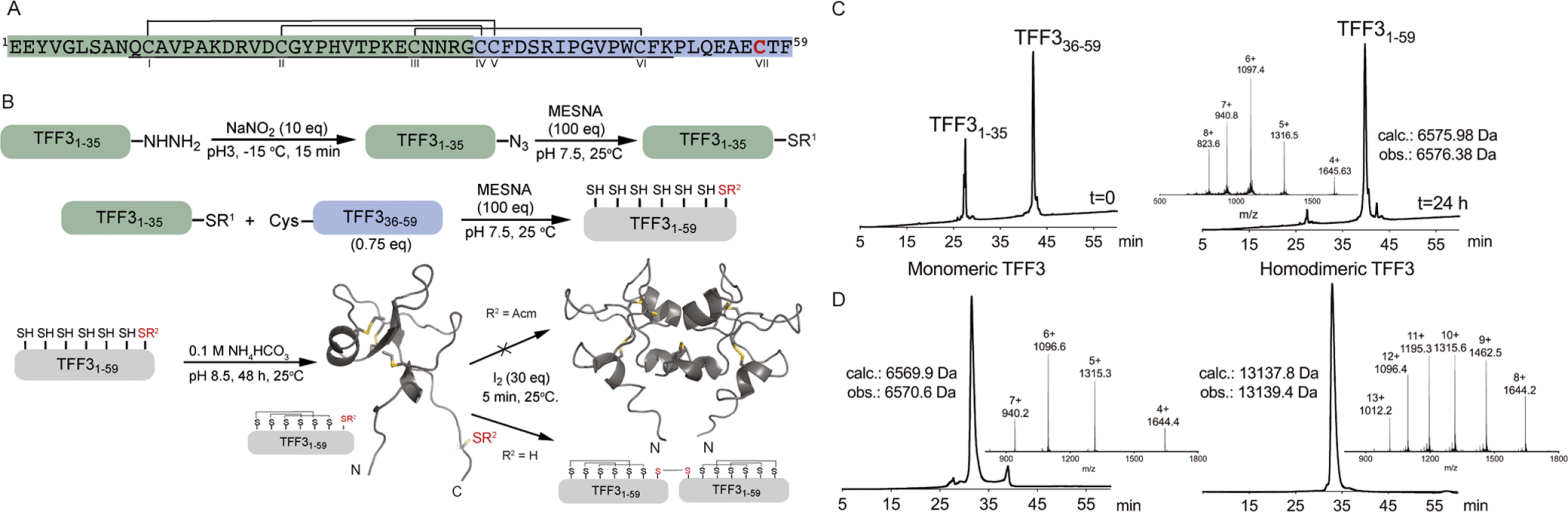
Synthesis of TFF3 and its homodimer. (A) TFF3 sequence and disulfide bond connectivity. Sequence highlighted in green and blue represents the *N*- and *C*-terminal fragments used for native chemical ligation, respectively, with Cys^57^ (red) used for dimerization. The trefoil domain is underlined. (B) Synthetic strategy used to produce TFF3 and its homodimer. Highlighted in gray is full-length TFF3. (C) Ligation reaction at time 0 h (left) and 24 h (right). (D) Analytical RP-HPLC chromatogram and MS of folded TFF3 monomer (left) and homodimer (right). TFF3 monomer displays a two-peak RP-HPLC profile due to its conformational complexity (see also Figure S3). TFF3 PDB: 1E9T; TFF3 homodimer PDB: 1PE3.

Single-chain assembly of full-length TFF3_1-59_ was not successful due to difficult sequence sections. We therefore switched to a two-fragment native chemical ligation (NCL) approach using the Fmoc-SPPS (9-fluorenylmethyloxycarbonyl-solid-phase peptide synthesis)-compatible hydrazide strategy (Figure 1).^50^ We split the TFF3 sequence into an *N*-terminal TFF3_1-35_ fragment with a glycine at the ligation site (faster ligation due to reduced steric hindrance) and a *C*-terminal TFF3_36-59_ fragment with an *N*-terminal cysteine residue at the ligation site (Figure 1A). We synthesized TFF3_1-35_ with a *C*-terminal hydrazide function (∼15% yield after purification) on a freshly prepared 2-Cl-(Trt)-NHNH_2_ resin and TFF3_36-59_ with a *C*-terminal acid (∼20% yield after purification) using a Phe-Wang PS resin. We activated TFF3_1-35_-NHNH_2_ with NaNO_2_ to form the *C*-terminal acyl azide followed by conversion of the azide into a thioester through addition of sodium 2-mercaptoethanesulfonate (MESNA) (Figure 1B).^50^ We then ligated the two fragments (TFF3_1-35_ and TFF3_36-59_) to produce the linear and fully reduced TFF3_1-59_ and purified it on a C_18_-RP-HPLC column (∼57% ligation yield) (Figure 1B,C).

After oxidative folding (0.1 M ammonium bicarbonate, pH 8.5, 48 h; Figure S1), we purified TFF3 on a C_5_-RP-HPLC column (Figure 1D, left). The TFF3 homodimer was produced *via* the formation of an intermolecular disulfide bond of unprotected Cys^VII^ (residue 57) through treatment with iodine (2 min) followed by C_5_-RP-HPLC purification (Figure 1D, right; 60% yield). Since dimerization of TFF3 via the unprotected Cys^VII^ residue was observed in aqueous solvents at pH > 7, we also synthesized a TFF3 analogue with Cys^VII^ protected with an acetamidomethyl (Acm) group (TFF3(C^57^Acm)) to prevent dimerization and to ensure clear functional distinction between monomeric and homodimeric TFF3 in further studies (Figure S2).

Folded TFF3, whether with Cys^VII^ protected or not, displayed a two-peak profile on analytical HPLC, with each peak having the correct mass (Figures 1D and S2). When these peaks were independently collected and reinjected, the same two-peak profile was observed (Figure S3), confirming that both peaks belong to TFF3. This is consistent with the conformational complexity of TFF3^45^ and an effect commonly observed with similarly complex peptides and proteins, including TFF1.^51−55^

### Synthetic TFF3 and TFF3 Homodimer Have the Correct Fold

**Figure 2:**
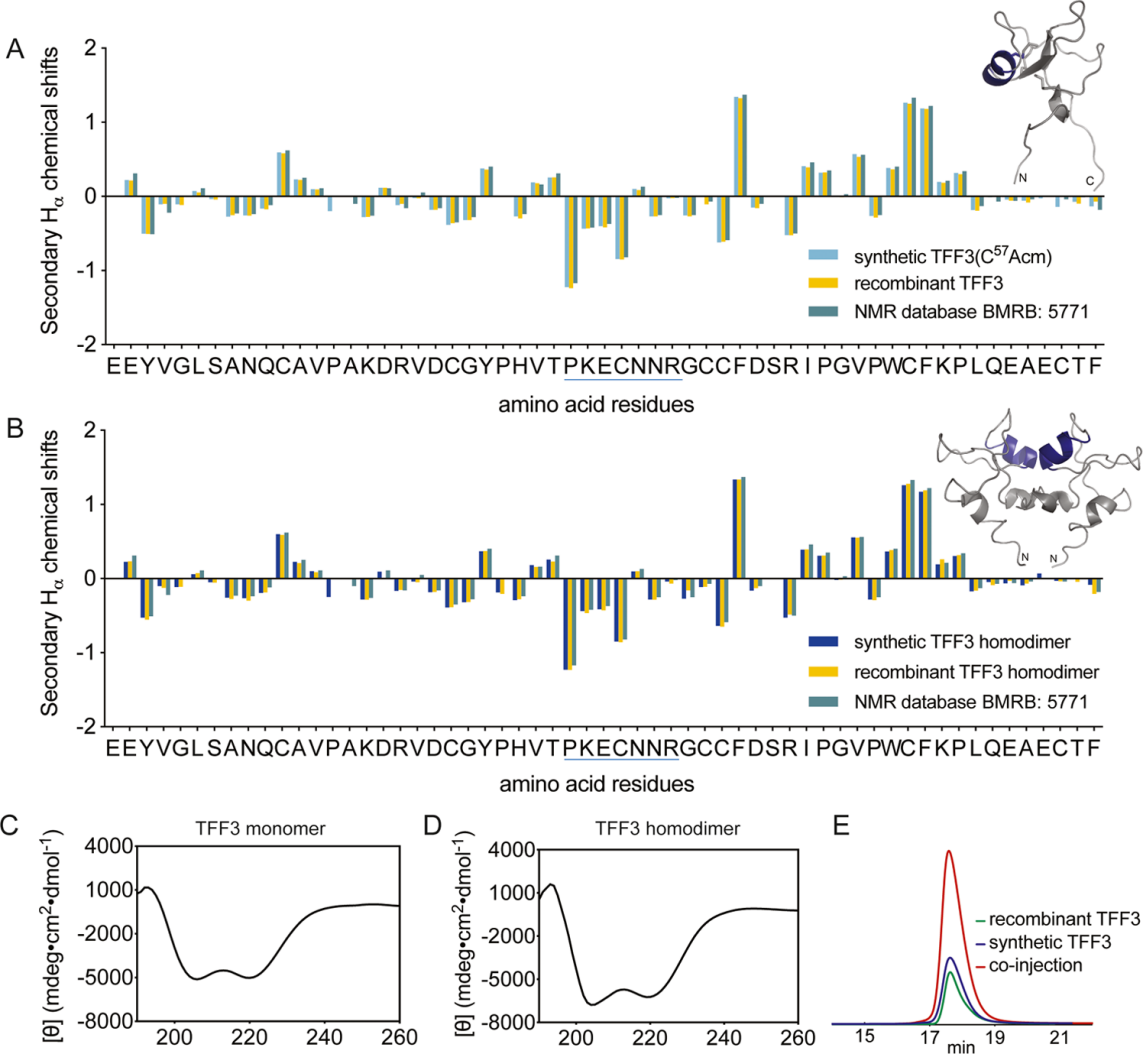
Comparison of secondary Hα chemical shifts of (A) TFF3(C^57^Acm) and (B) TFF3 homodimer produced by chemical synthesis with recombinant homologues and reported values from the Biological Magnetic Resonance Data Bank (BMRB: 5771). Secondary Hα chemical shifts were determined by subtracting the shifts observed in random coil peptides from the shifts determined from the two-dimensional (2D) NMR analysis.^57^ An α-helical region is highlighted in blue in the sequence and NMR structure. CD spectra of synthetic (C) TFF3(C^57^Acm) and (D) TFF3 homodimer. (E) Co-elution of synthetic and recombinant TFF3 homodimer (1:2 ratio) on a C_3_-RP-HPLC (1% gradient).

We characterized folded TFF3(C^57^Acm) and TFF3 homodimer by nuclear magnetic resonance (NMR) and circular dichroism (CD) experiments and compared them with the structures of recombinant TFF3 and TFF3 homodimer.^24,56^ The Hα chemical shifts of TFF3(C^57^Acm) and TFF3 homodimer were assigned using total correlated spectroscopy (TOCSY) and nuclear Overhauser effect spectroscopy (NOESY). Secondary chemical shifts were determined by subtracting random coil shifts from the Hα chemical shifts.^57^ Comparison of the secondary chemical shift of synthetic and recombinantly expressed TFF3 (Figure 2A), as well as those of the corresponding homodimers (Figure 2B), confirmed the correct fold.^24,56^ CD analysis of synthetic TFF3(C^57^Acm) indicated the presence of an α-helical structure characterized by negative bands at 222 and 208 nm and a positive band at 193 nm (Figure 2C,D). This aligned well with the structural information obtained from recombinant TFF3 provided by Dr. Lars Thim (Novo Nordisk A/S)^45^ that has a well-defined α-helix in loop 2. A co-elution study of synthetic and recombinant TFF3 homodimer further confirmed the NMR and CD results (Figure 2E).

### TFF3 and TFF3 Homodimer Reduce Apoptosis of Neuroblastoma Cells

**Figure 3:**
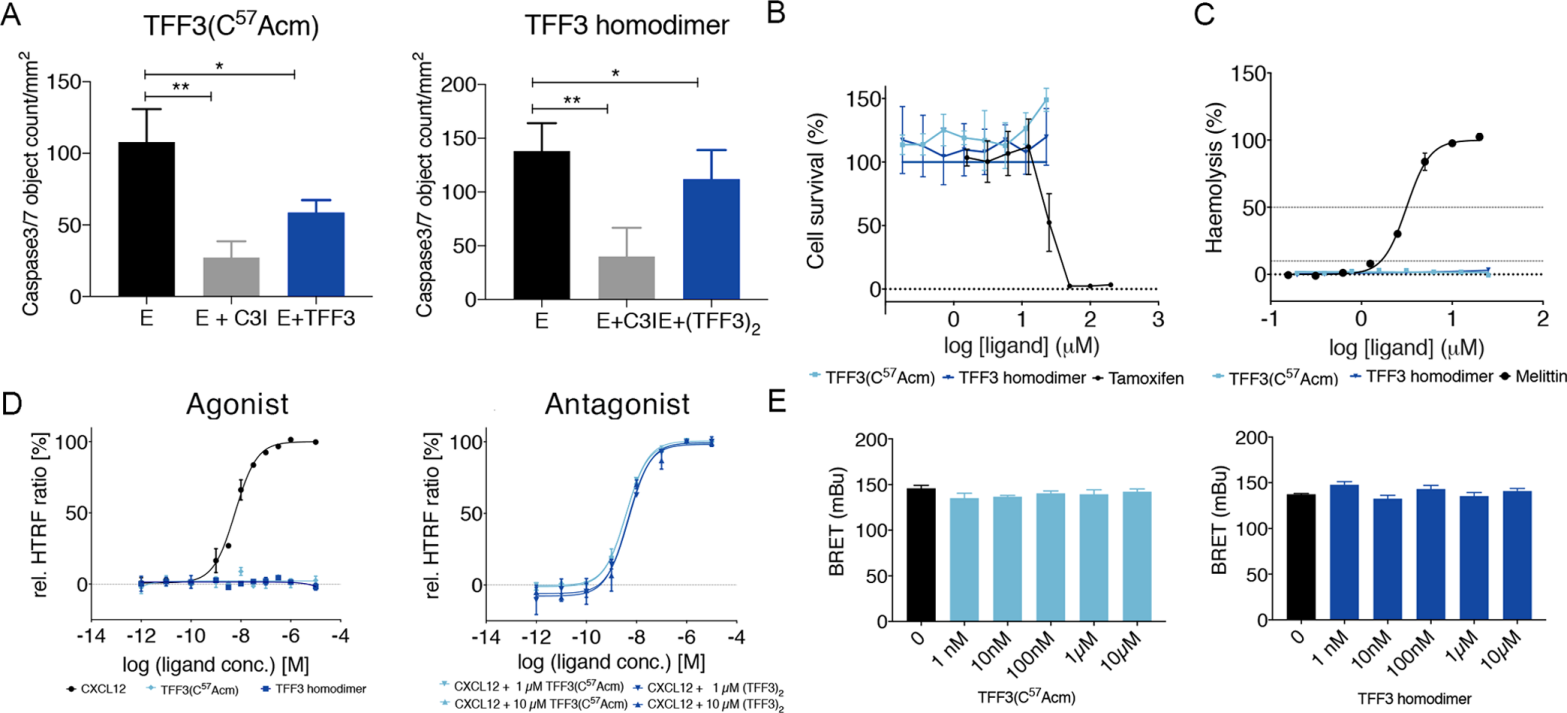
Biological characterization of TFF3(C^57^Acm) and TFF3 homodimer. (A) Cell death was induced with etoposide (10 μM), and cells were treated with 10 μM of TFF3. Etoposide and TFF3 were added at the same time. Caspase-3/7 activity was measured after 6 h. The results are expressed (mean ± standard error of the mean (SEM)) of *n* ≥ 3 independent experiments as the number of green fluorescent caspase-3/7 active objects generated by caspase-3/7 reagent added in media. Z-DEVD-FMK (50 μM; caspase-3 inhibitor) was used as the positive control. E: etoposide, E + C3I: etoposide + caspase-3 inhibitor. One-way analysis of variance (ANOVA) followed by Dunnett correction was performed to assess differences between treated cells and etoposide only. *p < 0.05, **p < 0.01. (B) TFF3 effect on the viability of HEK-293 cells after 20 h. Tamoxifen was used as a positive control for cell growth inhibition. Data are representative of two independent experiments shown as mean ± SEM. (C) Hemolytic activity of TFF3 after 1 h on erythrocytes. Melittin was used as a positive hemolytic control. Data are representative of two independent experiments shown as mean ± SEM. (D) Effect on CXCR4. Agonistic or antagonistic effects were assessed by the IP1 accumulation assay obtained through homogeneous time-resolved fluorescence (HTRF). CXCL12 was added for antagonistic studies after a 5 min preincubation of TFF3(C^57^Acm) or homodimer. For agonistic evaluation, all compounds were used individually for stimulation in the given concentration range.^58^ Neither TFF3(C^57^Acm) nor TFF3 homodimer were able to activate or inhibit the signal transduction of CXCR4 at concentrations up to 10 μM, indicating that these peptides are neither agonists nor antagonists at this receptor. The results are expressed as mean ± SEM of *n* ≥ 2 independent experiments. (E) Effect on LINGO2. HEK-293 coexpressing LINGO2-YFP and LINGO2-Rluc were stimulated with increasing amounts of TFF3(C^57^Acm) and TFF3 homodimer (1 nM–10 μM), but no significant increase in the bioluminescence resonance energy transfer (BRET) signal was observed. No ligand able to alter LINGO2 dimerization is known; therefore, no positive control could be used.^59^ Results are expressed as mean ± SEM for *n* = 4. One-way ANOVA followed by Dunnett correction was performed to assess differences between treated and nontreated cells.

Antiapoptotic activity of TFF3 was reported in cerebral ischemia.^22^ We therefore evaluated the capacity of TFF3(C^57^Acm) and TFF3 homodimer to reduce etoposide-induced cell death, by inhibiting caspase-3/7, in a neuroblastoma cell line (SH-SY5Y). TFF3(C^57^Acm) and TFF3 homodimer (10 μM) induced a statistically significant (*p* < 0.05) reduction in cell death (Figure 3A).

### TFF3 and TFF3 Homodimer Are Not Cytotoxic or Hemolytic

Considering the therapeutic potential of TFF3, it was important to determine any cytotoxic and hemolytic effects early on to avoid problems in later stages of drug development. We therefore assessed cytotoxicity on HEK-293 cells and hemolytic effects in human erythrocytes. We treated the cells with TFF3(C^57^Acm) and TFF3 homodimer and used resazurin, a blue dye that produces strong fluorescence when reduced by living cells, as the readout of the number of viable cells.^60^ In the hemolytic assay, we evaluated hemoglobin release, an indicator of erythrocyte lysis, upon exposure to the peptides. Neither TFF3(C^57^Acm) nor TFF3 homodimer displayed any cytotoxic or hemolytic effects at the concentrations of up to ∼25 μM (Figure 3B,C).

### TFF3 and TFF3 Homodimer Do Not Activate CXCR4 in COS-7 Cells Overexpressing the Receptor

CXCR4 is a member of the G protein-coupled receptor (GPCR) family^58^ and proposed as a target receptor for TFF3 to mediate wound healing.^30,39^ We therefore pharmacologically characterized TFF3(C^57^Acm) and TFF3 homodimer at CXCR4. We used a chimeric Gα_iq_ protein to switch the pathway to the Gα_q_ signaling, which leads to the activation of the phospholipase and allows measurement of CXCR4 activation through the production of inositol 1 phosphate (IP1).^61−63^ We measured IP1 accumulation upon stimulation with TFF3(C^57^Acm) and TFF3 homodimer in fibroblast-like COS-7 cells transiently transfected with CXCR4.^58^ This is a competitive immunoassay, based on the HTRF technology, where native IP1 produced by cells compete with labeled IP1 (acceptor) for binding to anti-IP1-cryptate (donor). The specific signal (i.e., Förster resonance energy transfer, FRET) is inversely proportional to the concentration of IP1 in the sample. The calculation of the fluorescence ratio eliminates possible medium interferences.^64^

TFF3(C^57^Acm) and TFF3 homodimer were tested alone or in combination with C-X-C motif chemokine 12 (CXCL12), the natural CXCR4 ligand,^58^ to assess TFF3’s potential to act as a CXCR4 agonist or antagonist. Neither TFF3(C^57^Acm) nor TFF3 homodimer activated CXCR4 at concentrations up to 10 μM in contrast to the positive control CXCL12 (EC_50_ 5.9 nM) (Figure 3D, left panel). The EC_50_ of CXCL12 was also not affected by TFF3 or TFF3 homodimer (up to 10 μM), suggesting that these peptides are also not antagonists (Figure 3D, right panel).

### TFF3 and TFF3 Homodimer Do Not Activate LINGO2 in BRET Assay

LINGO2 has also been put forward as a potential TFF3 receptor.^38^ We thus evaluated whether TFF3(C^57^Acm) or TFF3 homodimer could disturb LINGO2 dimerization *via* a bioluminescence resonance energy transfer (BRET) assay. This method allows the study of protein interactions using energy transfer between a light-emitting enzyme and a fluorescent acceptor protein.^65^ LINGO2 fused with *Renilla* luciferase (Rluc; protein donor) or yellow fluorescent protein (YFP; protein acceptor) were co-expressed in HEK-293 cells and BRET signal detected after adding the luminescent substrate coelenterazine. We observed a strong BRET signal under basal condition, demonstrating the capacity of LINGO2 to form dimers, as already described.^59^ We then evaluated whether the TFF3 constructs could promote changes of this basal BRET signal by inducing conformational change within the dimers and/or change the dimerization state of LINGO2. However, no statistically significant (*p* > 0.05) modification of BRET signal was observed following stimulation of HEK-293 cells coexpressing LINGO2-YFP and LINGO2-Rluc with TFF3(C^57^Acm) or TFF3 homodimer at any of the tested concentrations (0.1–10 μM) (*p* > 0.05) (Figure 3E).

### Gastrointestinal Stability Assays Revealed a Stable and Bioactive TFF3 Metabolite

TFF3 is often considered metabolically stable due to its rigid disulfide-rich structure and function in the gut.^3^ However, no systematic gut stability studies have been carried out and some reports indicate that TFF3 is not fully resistant to proteases. For example, only 15% of intravenously injected iodine-labeled TFF3 homodimer in rats was recovered from urine after 24 h.^26^ TFF3 homodimer was also degraded in the terminal parts of the large intestine^27^ and truncated at the C-terminal Phe^59^ in human saliva.^28^ It was thus important to characterize TFF3’s gastrointestinal stability in more detail, particularly considering that studies often rely on the use of sodium dodecyl sulfate (SDS) gels and antibodies to identify and characterize TFF3, where truncations can easily be missed.^48,49,66^

**Figure 4:**
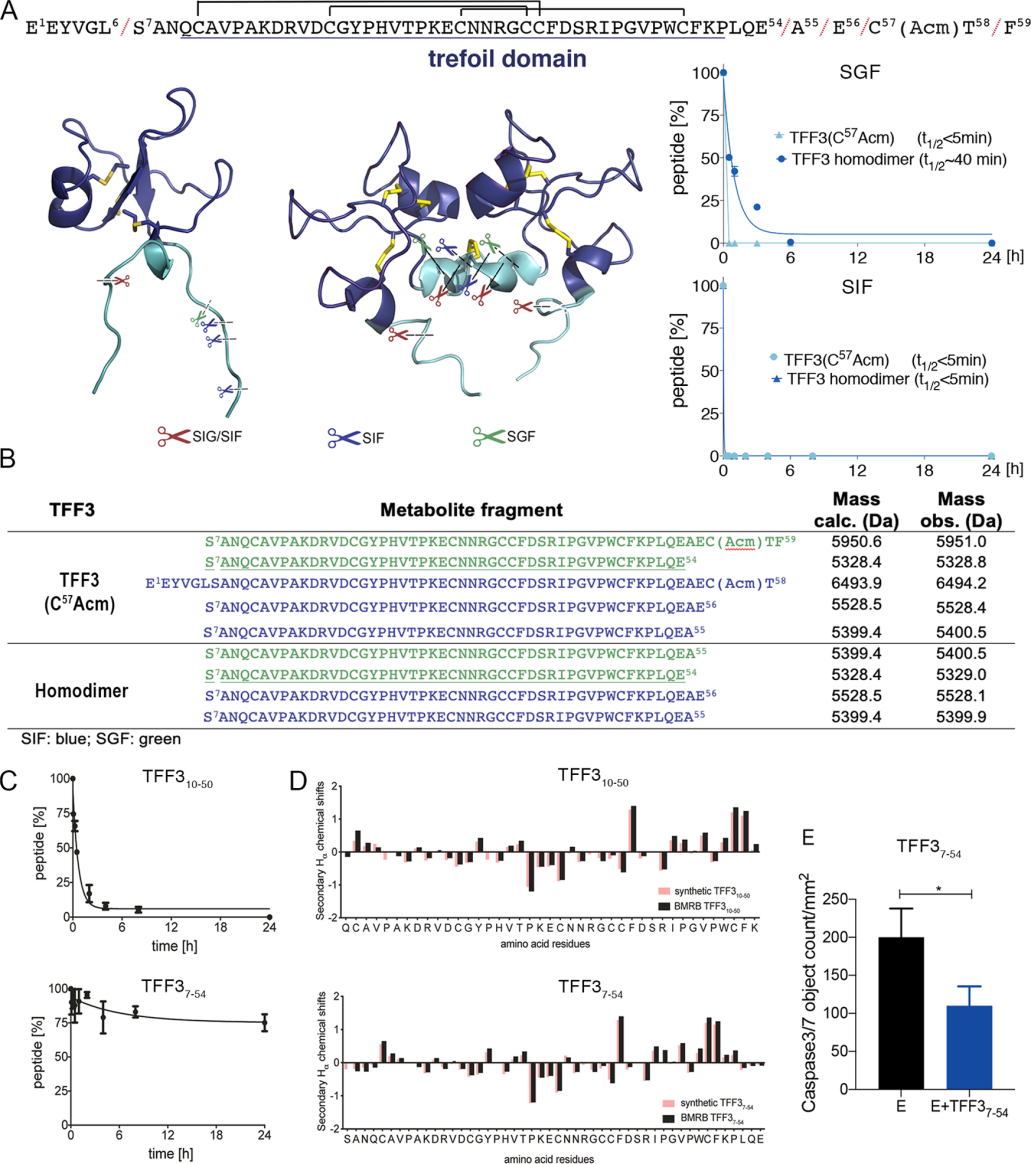
Identification and characterization of a stable and bioactive TFF3 metabolite (TFF3_7–54_). (A) The observed cleavage sites are highlighted in the three-dimensional structure and linear sequence of TFF3. The trefoil domain is underlined and displayed in blue in the sequence and NMR structure. (B) A table listing the observed fragments including their masses. The shortest stable metabolite (TFF_7-54_) is underlined. (C) Intestinal stability of the trefoil domain TFF3_10–50_ and the gut-stable metabolite TFF3_7–54_. (D) Comparison of the secondary Hα chemical shifts of TFF3 (BMRB: 5771) and the trefoil domain TFF3_10–50_, and TFF3 (BMRB: 5771) and the gut-stable metabolite TFF3_7–54_. Secondary Hα chemical shifts were determined by subtracting the shifts observed in random coil peptides from the shifts determined from the 2D NMR analysis.^57^ (E) Antiapoptotic effects of TFF3_7–54_ on SH-SY5Y cells. E: etoposide. Etoposide and TFF3_7–54_ were added at the same time. Caspase-3/7 activity was measured after 6 h. Results are expressed (mean ± SEM) of *n* ≥ 3 independent experiments as the number of green fluorescent caspase-3/7 active objects generated by caspase-3/7 reagent added in media. One-way ANOVA followed by Dunnett correction was performed to assess differences between treated cells and etoposide only. *p < 0.05, **p < 0.01.

We exposed TFF3(C^57^Acm) and TFF3 homodimer to simulated gastric fluid (SGF, containing pepsin, pH 1.3) and simulated intestinal fluid (SIF, containing pancreatic enzymes, pH 6.8), and monitored the mixture over 24 h by analytical RP-HPLC and MS. The *N*- and *C*-termini of TFF3 and its homodimer were readily truncated in both SGF and SIF (Figure 4A,B). In SGF, TFF3 was cleaved at Leu^6^/Ser^7^ followed by cleavage at Glu^54^/Ala^55^ (*t*_1/2_ < 5 min). In SIF, TFF3 was cleaved first at Thr^58^/Phe^59^ followed by cleavage at Leu^6^/Ser^7^, Glu^56^/Cys^57^, and Ala^55^/Glu^56^ (*t*_1/2_ < 5 min). TFF3 homodimer, in both SGF (*t*_1/2_ ∼ 40 min) and SIF (*t*_1/2_ < 5 min), was first broken down to monomeric TFF3 through simultaneous cleavage at Leu^6^ and Glu^54^ or Ala^55^, eventually leading to the same metabolites as TFF3 (Figure 4B). Importantly, we identified two metabolites (TFF3_7-54_ in SGF, and TFF3_7-55_ in SIF) that remained stable even after 24 h (Figure 4B).

In certain physiological environments, disulfide bonds are prone to scrambling or reductive cleavage, thereby affecting peptide integrity.^67^ Thus, we also evaluated the stability of the disulfide bonds of TFF3 in the presence of 10 equivalents of reduced glutathione (GSH) at pH 7 by time-course analytical RP-HPLC. No disulfide bond reduction or scrambling was observed for TFF3 or its homodimer (Figure S4) under these conditions. While this was expected for the relatively buried disulfide bonds within the trefoil domain (TFF3_10-50_), it highlights that also the disulfide bond outside of the trefoil domain is well protected.

We then synthesized the trefoil domain (TFF3_10-50_, single-chain assembly *via* Fmoc-SPPS) to investigate its gut stability. We acetylated the *N*-terminus (Gln^10^) of the trefoil domain to prevent the formation of pyroglutamic acid. TFF3_10-50_ was stable in SGF for 24 h but exhibited low stability in SIF (*t*_1/2_ < 30 min) (Figure 4C). We incubated TFF3_10-50_ with trypsin to identify some of the cleavage sites and observed cleavages at Lys^16^, Arg^18^, and Arg^41^ and fragments Glu^30^-Arg^34^ and Ile^42^-Lys^50^ linked by a disulfide bond. A comparison of the NMR secondary Hα chemical shifts of TFF3_10-50_ with those of full-length TFF3 (BMRB: 5771) indicated that removal of the *C*- and *N*-terminal tails outside the trefoil domain did not change TFF3’s overall domain structure (Figure 4D).

We also synthesized TFF3_7-54_ (single-chain assembly *via* Fmoc-SPPS) to confirm the initial stability results and to elucidate whether the residues outside the trefoil domain (S^7^AN^9^, P^51^LQE^54^) were responsible for its greater stability. TFF3_7-54_ was indeed stable in SGF and SIF with a half-life over 24 h (Figure 4C); ∼25% degradation was observed in SIF within 24 h and MS analysis identified that cleavage mainly occurred near the *C*-terminus (Leu^46^/Gln^47^ and Gln^47^/Glu^48^). Secondary Hα chemical shifts of TFF3_7-54_ confirmed that it had the same overall fold as full-length TFF3 (Figure 4D). TFF3_7-54_ was also active in the anti-apoptosis assay, equivalent to TFF3 and TFF3 homodimer (Figure 4E). Taken together, these results suggest that the *N*- and *C*-terminal extended domain residues in TFF3_7-54_ provide some steric protection against intestinal proteases and that the pharmacophore sits within the trefoil domain.

## Discussion

Reliable synthetic access to TFF3 and its homodimer has been a long-standing challenge due to its length, difficult sequence segments, disulfide-rich character, and the presence of a seventh unpaired cysteine residue (Cys^57^) that enables homo- or heterodimer formation.^1,30^ Here, we achieved the chemical synthesis of the monomers TFF3(C^57^Acm) and TFF3, and the TFF3 homodimer (Figure 1). TFF3(C^57^Acm) is a valuable TFF3 analogue that cannot dimerize under physiological conditions, thereby enabling a more controlled study of the effects of monomeric vs homodimeric TFF3. The synthesis was achieved *via* a combination of Fmoc-SPPS and two-fragment NCL followed by an efficient oxidative folding step using well-defined conditions (Figure 1). Compared to other approaches (i.e., recombinant expression), our strategy has the advantage of providing full control over site-specific chemical modifications, being compatible with combinatorial approaches and facilitating the incorporation of unnatural amino acids, bioconjugations handles, and reporter tags. This represents therefore an important new milestone for TFF3 research, since it considerably expands ligand design options, important for molecular probe development, structure–activity relationship (SAR) studies, and therapeutic lead development.

TFF3’s function has been associated with high metabolic stability to regulate gastrointestinal protection and repair, colorectal cancer development, and neuronal protection in the CNS.^1,3,30^ However, its mechanism of action and target receptors remain speculative and have not been independently validated^1,30^ and also the gastrointestinal stability has not been systematically investigated with some studies indicating that they are degraded.^26,28^ The presumed high gastrointestinal stability of TFF3^68−70^ would be an attractive feature from a peptide drug development point of view, since it could enable oral administration of TFF3-like drug candidates for the treatment of gastrointestinal disorders. With milligram quantities of TFF3 analogues at hand, we therefore investigated some of these aspects further to provide novel insights into TFF3’s metabolic stability and mechanisms of action.

TFF3 and its homodimer were rapidly enzymatically truncated at both termini in the gastric (TFF3 *t*_1/2_ < 5 min; TFF3 homodimer *t*_1/2_ ∼ 40 min) as well as in the intestinal (*t*_1/2_ < 5 min) environment (Figure 4A), revealing a gut-stable metabolite (TFF3_7-54_) (Figure 4D). The slightly shorter trefoil domain (TFF3_10-50_) degraded in the intestinal environment (*t*_1/2_ < 30 min) (Figure 4C), highlighting that the residues S^7^AN^9^ and P^51^LQE^54^ outside the trefoil domain are important for the protection against gastrointestinal degradation. The metabolite TFF3_7-54_ retained its overall three-dimensional structure, including its three loops and secondary motifs (Figure 4D), and displayed similar antiapoptotic activity as TFF3, suggesting that TFF3’s pharmacophore sits within the trefoil domain. TFF3 homodimer has been reported to be more potent than TFF3 in promoting cell motility,^31,32^ but considering the rapid degradation of the homodimer, it is remains questionable if it holds a major functional role in the gastrointestinal environment. This aligns with TFF3 being identified predominantly as a heterodimer (TFF3-FCGBP) in the colon, followed by the monomeric form, and only a small portion of TFF3 observed as a homodimer.^29^ None of the metabolites have so far been reported, which is not surprising considering that the methods to identify and characterize TFF3 (i.e., SDS gels and antibodies) can easily miss terminal truncations.^48,49^ The formation of heterodimers^29^ and TFF3 binding to mucins might furthermore protect against degradation.^71^

TFF3, TFF3 homodimer, and TFF3_7-54_ all reduced apoptosis of SH-SY5Y, a human neuroblastoma cell line (Figures 3 and 4), aligning well with reported antiapoptotic activity, including cerebral ischemia.^22,33,72,73^ These antiapoptotic effects support TFF3’s (neuro)protective role,^1,33,72,73^ as well as its association as a tumor growth promoter in different cancers.^8,74,75^ TFF3 and its homodimer did not display any cytotoxic or hemolytic effects, an important aspect for future therapeutic development of TFF3.

TFF3’s interaction with CXCR4, a member of the GPCR family,^58^ was proposed to mediate cell migration *via* an ERK1/2-independent signaling pathway.^39^ This interaction was established in a human conjunctival epithelial cell line expressing CXCR4, where blockage of CXCR4 impaired TFF3-mediated cell migration.^39^ While this suggests that CXCR4 is involved in the mechanism of action of TFF3, it did not provide evidence of direct TFF3-CXCR4 interaction. To validate this interaction, we tested TFF3 and TFF3 homodimer in a well-established CXCR4 signaling assay,^61−63^ demonstrating that they neither activated nor inhibited CXCR4 up to a concentration of 10 μM (Figure 3D). These results correspond with another study that failed to co-localize TFF3 and CXCR4.^38^

The second putative TFF3 receptor that we investigated was LINGO2, which has recently been implicated with TFF3 in promoting protection against colitis *in vivo.*(38) Neither TFF3 nor TFF3 homodimer displayed any effect on LINGO2 dimerization, which was assessed by BRET (Figure 3E).^59^ Due to the limited availability of functional LINGO2 bioassays, we cannot fully exclude that TFF3 does not signal through LINGO2 and we can only state that TFF3 does not interfere with LINGO2 dimerization.

## Conclusions

We have developed reliable synthetic strategies to produce TFF3 and analogues, which will markedly facilitate mechanistic and SAR studies as well as therapeutic development. We demonstrated that TFF3, TFF3 homodimer, and the trefoil domain (TFF3_10-50_) are readily degraded in the gastrointestinal environment, revealing We were not able to pharmacologically confirm a TFF3 signaling or interaction with CXCR4 or LINGO2. The chemical synthesis of TFF3 and its homodimer as well as the discovery of the truncated bioactive TFF3 metabolite are important new developments for the field that provide new perspectives and opportunities for the design and development of advanced molecular probes and TFF3 analogues facilitating both fundamental research as well as therapeutic development.

## Experimental Section

### Materials

Fmoc-amino acids and Fmoc-Phe-Wang Tenta Gel resin (loading 0.7 mmol/g) were purchased from Iris Biotech GmbH (Marktredwitz, Germany). Rink amide Protide resin (loading 0.19 mmol/g) and Oxyma Pure (ethyl cyanohydroxyiminoacetate) were obtained from CEM (Charlotte, NC, USA). 2-Chlorotrityl chloride resin (loading 2.0 mmol/g) was from Chem-Impex (Wood Dale, IL, USA). Pepsin from porcine gastric mucosa (3500–4500 units/mg solid), hydrazine hydrate and recombinant EGF (epidermal growth factor), and *N*,*N*′-diisopropylcarbodiimide (DIC) were from Sigma-Aldrich (Sydney, Australia). *N*,*N*-Dimethylformamide (DMF), pancreatin from porcine pancreas, trifluoroacetic acid (TFA), and diethyl ether were obtained from Chem-Supply (Gillman, Australia). Trypsin-EDTA 0.25%, Dulbecco’s modified Eagle’s medium (DMEM), and l-glutamine were from Invitrogen (Mulgrave, Australia). Fetal bovine serum (FBS) was from Scientifix (South Yarra, Australia). IncuCyte caspase-3/7 green apoptosis reagent was purchased from Essenbioscience (Newark Close, U.K.). HEK-293 (ATCC CRL-1573) human embryonic kidney and SH-SY5Y cells were obtained from American Type Culture Collection (ATCC). Chitin beads were purchased from New England Biolabs GmbH (Frankfurt, Germany). IP-One Gq assay kit from CisBio (Codolet, France). Metafectene Pro was from Biontex Laboratories GmbH (Munich, Germany). pcDNA3.1 plasmid was kindly provided by Dr. Evi Kostenis, Rheinische Friedrich-Wilhelms-Universität, Bonn, Germany. All solvents were obtained in the highest available purity and used without further purification. All other chemicals were obtained from Sigma-Aldrich/Merck (Sydney, Australia) in the highest available purity. Recombinant monomeric and homodimeric human TFF3 produced in yeast were kindly provided by Dr. Lars Thim (Novo Nordisk A/S, Denmark). Human whole blood was obtained from the Australian Red Cross Blood Service.

### Ethics Statement

Human ethics approval was obtained for use of human blood for hemolysis studies, from the University of Queensland Medical Research Ethics Committee (approval number 2014000031).

### Peptide Synthesis

#### Preparation of 2-Chlorotrityl Hydrazine Resin

2-Chlorotrityl chloride resin was swelled in 50% DMF/DCM (v/v) for 30 min in a peptide synthesis vessel. The solution was drained, and the resin was treated with 10% hydrazine hydrate/DMF (v/v) for 30 min. After draining the solution, the resin was washed with DMF. Unreacted resin was capped with 5% MeOH/DMF (v/v) for 10 min and washed with DMF. The resin was directly used for the next coupling step. Resin loading was determined by quantitative Fmoc release. Briefly, 20% piperidine/DMF was added to a 10 mL volumetric flask containing 10 mg of dry resin and mixed for 30 min. A UV cuvette was filled with 100 μL of the supernatant and diluted 1:10 with 20% piperidine/DMF. The absorbance of the dibenzofulvene-piperidine adduct was measured using a UV spectrometer at 301 nm. The resin loading (mmol/g) was then calculated using the following formula: *A*/(e × *d* × *m*) × 106, where *A* is the absorbance, *e* is the extinction coefficient of dibenzofulvene adduct, *m* is the mass of resin (mg), and *d* is the dilution factor.

#### Solid-Phase Peptide Synthesis

NCL precursor peptide fragments of TFF3 (Cys^57^(Acm) and free Cys^57^ forms) were synthesized on a Liberty Prime automatic synthesizer (CEM, Charlotte, NC, USA) *via* Fmoc-SPPS on a 0.1 mmol scale. C-terminal fragment was synthesized using Fmoc-Phe-Wang (TFF3_36-59_) and the hydrazide fragment (TFF3_1-35_-NHNH_2_) on a freshly prepared 2-chlorotrityl hydrazide resin. TFF3_10-50_ and TFF3_7-54_ were synthesized on a Rink amide Protide resin. Amino acid side chains were protected as follows: Arg(2,2,4,6,7-pentamethyldihydrobenzofuran-5-sulfonyl), Asn/Gln(trityl), Asp(O-3-methylpent-3-yl), Glu(*tert*-butyl ester), Cys(trityl or acetamidomethyl), His/Lys/Trp (*tert*-butyloxycarbonyl), and Ser/Thr/Tyr(*tert*-butyl). Fmoc deprotection was performed using 25% pyrrolidine/DMF. Couplings (5 equiv) were carried out with DIC/Oxyma Pure at 105 °C. Fmoc-amino acid/DIC/Oxyma (1:2:1). Upon completion of the peptide chain, the resin was washed with DCM/MeOH. Cleavage from the resin and simultaneous removal of side-chain-protecting groups was achieved by treatment with 90% trifluoroacetic acid (TFA)/5% triisopropylsilane (TIPS)/5% H_2_O at 25 °C for 90 min. Following cleavage, the solution was evaporated under a stream of N_2_ and the products precipitated and were washed with cold Et_2_O and lyophilized in 50% acetonitrile (ACN)/0.1% TFA/H_2_O. The crude products were purified by preparative HPLC.

#### Native Chemical Ligation

Only fragments with purity >90% were used for the NCL. The N-terminal fragment containing the hydrazide group (1.5 mM) was oxidized to an azide by dissolving the peptide in 0.2 M sodium phosphate buffer solution containing 6 M Gn·HCl (pH 3) and reacting it with NaNO_2_ (10 equiv relative to the hydrazide fragment) for 15 min at −15 °C. During this step, the C-terminal fragment (1 mM) was dissolved in 0.2 M phosphate solution containing 6 M Gn·HCl and sodium 2-mercaptoethanesulfonate (MESNA; 100 equiv relative to the hydrazide fragment). The solutions containing the peptide segments were combined, and the pH was carefully adjusted to 7.5 with NaOH. The reaction was monitored by analytical RP-HPLC and carried out for 24 h and purified by preparative RP-HPLC. TCEP (75 mM) was added to the reaction before RP-HPLC analysis and purification.

#### Oxidative Folding

Peptides were dissolved in a minimal amount of 50% ACN/0.1% TFA/H_2_O and added to the oxidative buffer (0.1 M NH_4_HCO_3_) for a final concentration of 50 μM, and the pH was adjusted to 8.5. Oxidation was monitored by analytical RP-HPLC and electrospray mass spectroscopy (ESI-MS). After complete oxidation, the pH was adjusted to 2 with neat TFA, filtered, and the peptide was purified by preparative RP-HPLC.

#### Dimerization

Folded monomeric TFF3 with the Cys^57^ unprotected was dissolved in 50% ACN/0.1% TFA/H_2_O to a final concentration of 2.5 mM. Iodine (30 equiv) was added to accelerate the dimerization. After complete oxidation (2 min), the reaction was quenched with ascorbic acid and the peptide was purified by preparative RP-HPLC (∼60% yield).

#### RP-HPLC and LC-MS Methods

Peptides were purified using either a preparative C_18_ (Grace Vydac; 10 μm, 2.2 cm ID × 250 mm, flow rate 15 mL/min) or C_5_ (Phenomenex Luna; 10 μm, 21.2 mm ID × 250 mm, flow rate 15 mL/min) columns on a Waters 600 HPLC system (Waters Co., Milford, MA, USA) using gradient of solvent A (0.05% TFA in water) and B (90% ACN/0.043% TFA/10% H_2_O) according to the peptide retention time observed by analytical RP-HPLC. TFF3_1-35_ was purified using the C_18_ preparative RP-HPLC column with a gradient of 10–40% B over 60 min (15% yield). TFF3_36-59_ (20% yield) and reduced TFF3 (59% ligation yield) were also purified on the C_18_ preparative column with a gradient of 20–50% B over 60 min. Reduced TFF3 was washed with 10% B for 15 min before its purification to remove the salts from the ligation buffer. Folded TFF3 was purified using the C_5_ preparative RP-HPLC column with a gradient of 10–40% B over 60 min. The molecular mass of the fractions collected was analyzed by direct injection in an ESI-MS, and those with the desired mass were further analyzed by RP-HPLC and lyophilized. Peptides were analyzed by RP-HPLC using an analytical C_3_ (Agilent Zorbax SB-C_3_, 5 μm, 2.1 mm × 250 mm, 300 Å) or C_18_ (Phenomenex Jupiter; 5 μm, 2.1 mm × 250 mm, 300 Å) RP-HPLC column connected to a Shimadzu LC-20AT solvent delivery system equipped with an SIL-20AHT autoinjector and an SPD-20A Prominence ultraviolet–visible detector. Data were recorded and processed with the Shimadzu LabSolutions software (version 5.90). A linear gradient from 0–60% solvent B in 60 min was performed, and absorbance data were collected at 214 nm to determine the purity of the final product. Only peptides with purity >95% were used for structural and biological analyses. The mass analysis of the peptides was performed using a Q-Star Pulsar mass spectrometer (SCIEX, Ontario, Canada) with a Series 1100 solvent delivery system equipped with an autoinjector (Agilent Technologies, Inc., Palo Alto, CA) and a Phenomenex Jupiter LC-MS C_18_ column (90 Å, 4 μm, 2 mm × 250 mm). Linear gradients of 0.1% aqueous formic acid (solvent A) and 90% ACN/0.1% formic acid (solvent B) were employed at a flow rate of 250 μL/min, and the column was maintained at 45 °C. The instrument was scanned in the *m*/*z* range of 500–1800 Da. Data acquisition and processing were carried out using Analyst software v1.1 (SCIEX, Canada).

#### Co-elution RP-HPLC Study of TFF3 Homodimer

Synthetic and recombinant homodimeric TFF3 were co-injected at a 2:1 (synthetic:recombinant) ratio and subjected to analytical RP-HPLC analysis using a 1% solvent B/min gradient on a C_3_-RP-HPLC column (Agilent Zorbax SB-C_3_, 5 μm, 2.0 mm × 250 mm, 300 Å).

#### *In Vitro* Stability Assay

Simulated gastric fluid (SGF) was prepared by dissolving 20 mg of NaCl and 8 mg of pepsin in 70 μL of concentrated HCl (32%), and the volume was diluted to 10 mL with Milli-Q water (pH 1.3).^76,77^ Simulated intestinal fluid (SIF) was prepared by dissolving 68 mg of KH_2_PO_4_ in 500 μL of Milli-Q water followed by the addition of 800 μL of 0.2 M NaOH and 100 mg of porcine pancreatin, and the volume was adjusted to 10 mL with Milli-Q water (pH 6.8).^76,77^ Peptide stock solution (1 mM; 15 μL) was added to SGF (285 μL) or SIF (285 μL) and incubated at 37 °C. Samples (30 μL) from SGF and SIF were taken at 0, 5, 15, and 30 min and 1, 2, 4, 8, and 24 h timepoints and subsequently quenched with 30 μL of 0.2 M Na_2_CO_3_ (SGF) or 30 μL of 5% aqueous TFA (SIF). The samples were analyzed by analytical RP-HPLC (30 μL) and/or LC-MS (20 μL). The amount of peptide remaining was determined by measuring the peak area and expressing it as a % of the peak area at time 0. Peptide half-life (*t*_1/2_) was determined from the peptide degradation profiles using an exponential one-phase decay fit in Prism (version 7, GraphPad, La Jolla). LC-MS analysis was performed in a Q-Star Pulsar mass spectrometer (SCIEX, Ontario, Canada), and the raw data spectra were processed using the peptide reconstruction tool in the BioAnalyst software to identify the mass of the metabolites. The stability of the disulfide bonds was assessed through incubation of the peptides (10 μM) with 10 equiv of reduced glutathione (GSH; 100 μM) in sodium phosphate (50 mM) buffer at pH 7.2. Samples were taken at timepoints 0, 4, 8, and 24 h, quenched by adding 10% TFA, and analyzed by analytical RP-HPLC using an analytical C_3_ column (Agilent Zorbax SB-C_3_, 5 μm, 2.1 mm × 250 mm, 300 Å).

#### Circular Dichroism

Stock solutions of the peptides were prepared in 50% ACN/H_2_O at 1 mM concentration. Peptide concentrations for CD analysis were 50 μM in 10 mM sodium phosphate buffer (pH 7.4). CD spectra were obtained on a Jasco J-810 spectropolarimeter (Easton, MD). All experiments were carried out in a 0.1 cm quartz cell with 250 μL of sample at 25 °C and examined in the far-UV spectra region (185–260 nm), 20 nm/min scanning speed, 1 nm bandwidth, and 0.5 nm data pitch with five scans averaged for each sample. Blank subtraction was performed in the Spectra Management Software followed by smoothening using the Savitzky–Golay method. CD was reported as mean residue ellipticity ([θ] (mdeg·cm^2^·dmol^–1^) = (100 × θ)/(*n* × *c* × *l*), where θ is the raw output (mdeg), *n* is the number of peptide bonds, *c* is the concentration (M), and *l* is the cuvette path length (cm)).

#### Nuclear Magnetic Resonance

NMR spectra of peptides dissolved in 90% H_2_O/10% D_2_O (∼1 mM) were recorded using a Bruker 600 MHz Avance III NMR spectrometer equipped with a cryogenically cooled probe (cryoprobe) at 298 K. NOESY spectra was recorded with a mixing time of 200 ms and TOCSY with spin lock of 80 ms. Samples were internally referenced to water at 4.76 ppm. TopSpin (Bruker Biospin) and CCPNMR Analysis 2.4.1 (CCPN, University of Cambridge, Cambridge, U.K.) were used to process and assign the spectra, respectively. NOEs in the NOESY spectrum were manually picked and assigned. Secondary shifts were calculated by subtracting the random coil Hα shift from the experimental Hα shifts.^57^

#### Antiapoptotic Assay

SH-SY5Y cells were seeded (13–15 × 10^3^ cells/well) in 96-well microplates in DMEM with 10% (v/v) fetal calf serum (FCS; Gibco), 2 mM L-glutamine, and 100 units/mL penicillin/streptomycin. The media was replaced with low riboflavin, complete Ham’s F12 medium with 10% (v/v) fetal calf serum, 2 mM L-glutamine, and 100 units/mL penicillin/streptomycin before the live-cell imaging. Cells were treated with the etoposide or the caspase-3 inhibitor or TFF3(C^57^Acm) or TFF3 homodimer in the presence of IncuCyte caspase-3/7 green apoptosis reagent. Data were acquired using an IncuCyte ZOOM instrument with standard scan type setting (4 images per well) every 2 h for 48 h.

#### Cell Viability/Cytotoxicity Assays

HEK-293 cells (5000 cells/well), suspended in DMEM supplemented with 10% FBS, were seeded into 384-well plates in a volume of 20 μL. Monomeric and homodimeric TFF3 were added to the cells for a final concentration of 0.18–22.7 μM. The cell plates were then incubated for 20 h at 37 °C and 5% CO_2_. Tamoxifen was used as a positive control. After the incubation, 5 μL of 100 μM resazurin diluted in PBS was added to each well (final concentration ∼11 μM). The plates were then incubated for 3–4 h at 37 °C and 5% CO_2_. The fluorescence intensity (FI) was read using the TECAN Infinite M1000 PRO with excitation/emission 560/590 nm. Cytotoxicity or cell viability was calculated using the following equation: cell viability (%) = (FI_sample_ – FI_negative_/FI_untreated_ – FI_negative_) × 100. CC_50_ (concentration at 50% cell viability) was calculated using a nonlinear regression analysis of log (concentration) vs normalized cell viability.

#### Hemolysis Analysis

Monomeric and homodimeric TFF3 were serially diluted twofold in 0.9% NaCl and seeded (25 μL) in a 384-well polypropylene plate (0.2–25 μM final concentration). Whole blood (10 mL/tube) was washed two to three times in three volumes of 0.9% NaCl, with centrifugation of 500*g*, with reduced deceleration, for 10 min between washes. The cells were counted using a Neubauer hemocytometer and then diluted to 1 × 10^8^/mL in 0.9% NaCl. The cells (25 μL/well) were added to the plates containing TFF3. Melittin was used as a positive control. The plates were sealed and then placed on a plate shaker for 10 min before being incubated for 1 h at 37 °C without shaking. Following incubation, the plates were centrifuged at 1000*g* for 10 min to pellet cells and debris and then 25 μL of the supernatant was transferred into a 384-well flat-bottom PS plate and absorbance (Abs) was read at 405 nm using a Tecan M1000 Pro monochromator plate reader. Percent hemolysis was calculated using the following equation: Hemolysis (%) = (Abs_sample_ – Abs_negative_/Abs_positive_ – Abs_negative_) × 100. HC_10_ and HC_50_ (concentration at 10 and 50% hemolysis, respectively) were calculated using nonlinear regression analysis of log (concentration) vs normalized hemolysis.

#### Recombinant Expression and Purification of Human CXCL12

The human CXCL12 (natural CXCR4 ligand) cDNA was cloned into the pTXB1 vector *via* Nde I and Sap I restriction sites. Generated plasmids were amplified in *E. coli* DH5α in Luria-Bertani (LB) medium with 100 μg/mL ampicillin and purified by the PureYield plasmid miniprep system (Promega GmbH, Mannheim, Germany). The correctness of the generated constructs was verified by Sanger dideoxy sequencing of the entire CXCL12 sequence. CXCL12 was expressed as fusion protein with a small intein domain from the *Mycobacterium xenopigyrA* gene and a chitin-binding domain (CBD) in *E. coli* ER2566 in LB medium containing 100 μg/mL ampicillin for 5 h at 37 °C under shaking. Notably, the initial methionine, which is not present in the mature human protein, is not cleaved by *E. coli*; thus, the generated protein bears an additional *N*-terminal methionine.^78^ After cell lysis, and inclusion body extraction and solubilization, fusion protein was purified on chitin beads and target protein was eluted with column buffer (20 mM HEPES, 500 mM NaCl, 1 mM EDTA, 3 M urea, pH 8 at 4 °C) containing 0.1 M DTT (dithiothreitol) and 0.2% Tween-20, according to the manufacturer′s protocol. The protein thioester was subsequently hydrolyzed under basic conditions at pH 10 and 4 °C, and the target protein was purified by preparative RP-HPLC on a Phenomenex Jupiter C_18_ column (300 Å, 5 μm, 250 mm × 21.2 mm) using linear gradients of 0.1% TFA/H_2_O and 0.08% TFA/ACN. The protein was restored in 0.1 M NaH_2_PO_4_, 6 M guanidine hydrochloride, pH 6.0, and refolded by rapid dilution as described earlier.^79^ Finally, refolded protein was isolated *via* preparative RP-HPLC on a Phenomenex Jupiter C_18_ column (300 Å, 5 μm, 250 mm × 21.2 mm) applying linear gradients of 0.1% TFA/H_2_O and 0.08% TFA/ACN. Identity and purity were determined with ESI-/MALDI-ToF mass spectrometry (Bruker Daltonik GmbH, Bremen, Germany) and analytical RP-HPLC, respectively. The protein concentration was determined by photometric measurement at 280 nm using the corresponding extinction coefficient.

#### Inositol 1 Phosphate (IP1) Assay

COS-7 cells (fibroblast-like cells from African green monkey) were transiently co-transfected with the CXCR4 *C*-terminally fused to eYFP in pVitro2 vector and the untagged chimeric G protein Gα_Δ6qi4myr_ in pcDNA3.1 plasmid using Metafectene Pro according to manufacturer’s protocol. The cells were cultured in DMEM with higher glucose and supplemented with 10% FCS without any antibiotics at 37 °C and 5% CO_2_ in 95% humidity. Cisbio IP-One G_q_ assay kit was used according to previous description with minor modifications to measure activity.^80^ Briefly, a standard curve was prepared in HBSS (Hanks’ balanced salt solution) with 20 mM LiCl to determine the linear range of the assay and 10 000 cells/well were cultured overnight in a 384-well flat white plate (Greiner Bio-one GmbH, Frickenhausen, Germany). TFF3 and its homodimer as well as CXCL12 were diluted in HBSS containing 20 mM LiCl. Stimulation was carried out for 1 h in triplicate at 37 °C. Subsequently, 3 μL of IP1-d2 and 3 μL of Ab-cryptate in lysis buffer were added to the wells and incubated on a tumbler for 60 min at 25 °C. Fluorescence was then measured at 620 and 665 nm and the HTRF (homogeneous time-resolved fluorescence) ratio (665/620 nm) was calculated. Data analysis was performed with Prism (version 7, GraphPad, La Jolla). Tested compounds were normalized to CXCL12 wild type, with the highest HTRF ratio set to 0% and the lowest HTRF ratio set to 100% response. For testing the agonistic activity TFF3(C^57^Acm), TFF3 homodimer and CXCL12 were individually used for stimulation in the concentration range of 10^–12^ to 10^–5^ M. Antagonistic activity was tested by stimulation of the cells with CXCL12 in the concentration range of 10^–12^ to 10^–5^ M after preincubation for 5 min with 1.5-fold concentrated TFF3 or its homodimer, respectively. The final concentration range of 10^–12^ to 10^–5^ M to CXCL12 and 1 or 10 μM to TFF3(C^57^Acm) and TFF3 homodimer was achieved by adding the compounds from a threefold concentrated stock solution.

#### Bioluminescence Resonance Energy Transfer (BRET) Assay

HEK-293 cells were cultured with 10% FBS at 37 °C and 5% CO_2_ in DMEM for 72–96 h. The cells were passaged to collagen-pretreated 10 cm plates (1/200 dilution) and incubated at 37 °C and 5% CO_2_ overnight. The medium was changed 1 h before transfection. BRET assays were performed following transient transfection of the donor protein (LINGO2-RLuc) alone or with the acceptor protein (LINGO2-YFP) using the calcium phosphate method. The cells were rinsed twice with sterile PBS 1× and incubated in fresh DMEM with 10% FBS at 37 °C and 5% CO_2_ overnight 1 day after transfection. Following 48 h of transfection, the medium was removed and the cells were rinsed twice with PBS 1× and harvested with 10 mL of HBSS at 25 °C. The cell suspension was seeded in quadruplets in a 96-well plate (∼10 000 cells/well) in which different concentrations of monomeric and homodimeric TFF3 have been loaded. After 20 min incubation, the fluorescence was measured on a Mithras LB 940 Multimode Microplate Reader (Berthold Technologies, Germany) to check if the peptides alone did not modify fluorescent emission of YFP following excitation at 485 nm and reading at 530 nm. Then, total luminescence was measured by following coelenterazine addition (final concentration 5 μM). BRET signal was measured in four repeats and calculated by determining the emission ratio at 530/480 nm on cells coexpressing donor and acceptor and by subtracting the emission background BRET signal ratio (530/480 nm) of cells expressing only donor protein, then multiplying by 1,000 to obtain results in millBRET units (mBU).

## Supporting Information Available

Oxidative folding of TFF3, analytical HPLC and high-resolution MS traces of TFF3(C^57^Acm) chemical synthesis, analytical HPLC profile of TFF3, and stability of TFF3 disulfide bonds (PDF)
Molecular formula strings (CSV)

## Abbreviations

BRET: bioluminescence resonance energy transfer
CD: circular dichroism
CXCL12: C-X-C motif chemokine 12
CXCR4: chemokine receptor type 4
CNS: central nervous system
EGFR: epidermal growth factor receptor
FCGBP: Fc fragment of IgG Fc binding protein
Fmoc-SPPS: 9-fluorenymethyloxycarbonyl-solid-phase peptide synthesis
GSH: reduced glutathione
HEK-293: human embryonic kidney 293
IBD: inflammatory bowel diseases
LINGO2: leucine-rich repeat receptor and nogo-interacting protein 2
MESNA: sodium 2-mercaptoethanesulfonate
NOESY: nuclear Overhauser effect spectroscopy
NMR: nuclear magnetic resonance
SGF: simulated gastric fluid
SIF: simulated intestinal fluid
TOCSY: total correlation spectroscopy
