# Supplementary material for: Chemical Synthesis of TFF3 Reveals Novel Mechanistic Insights and a Gut-Stable Metabolite
Source: J Med Chem. 2021 Jun 18;64(13):9484–95. doi: 10.1021/acs.jmedchem.1c00767 (PMC8273887; doi:10.1021/acs.jmedchem.1c00767)
Supplement: Supplementary file 1 — jm1c00767_si_001.pdf [file jm1c00767_si_001.pdf]

Supporting Information

Chemical Synthesis of TFF3 Reveals Novel Mechanistic Insights
and a Gut-Stable Metabolite

Nayara Braga Emidio[1], Rajeshwari Meli[2], Hue N. T. Tran[1], Hayeon Baik[2], Séverine Morisset-Lopez[3], Alysha G. Elliott[1], Mark A. T. Blaskovich[1], Sabrina Spiller[4], Annette G. Beck-Sickinger[4], Christina I. Schroeder[1,5], Markus Muttenthaler[1,2*]

[1] Institute for Molecular Bioscience, The University of Queensland, Brisbane, QLD 4072, Australia.

[2] Institute of Biological Chemistry, Faculty of Chemistry, University of Vienna, 1090 Vienna, Austria.

[3] Centre de Biophysique Moléculaire, CNRS, Unité Propre de Recherche 4301, Université d'Orléans, 45071 Orleans, France

[4] Institute of Biochemistry, Faculty of Life Sciences, Leipzig University, Leipzig, 04103, Germany.

[5] Center for Cancer Research, National Cancer Institute, National Institutes of Health, Frederick, MD, 21702, USA.

*Correspondence: markus.muttenthaler@univie.ac.at (M. Muttenthaler).

Table of Content

Figure S-1. Oxidative folding of TFF3 S3

Figure S-1. Chemical synthesis of TFF3(C^{57}Acm) S3

Figure S-2. Analytical HPLC profile of TFF3 S4

Figure S-3. Stability of TFF3 disulfide bonds S4

Results

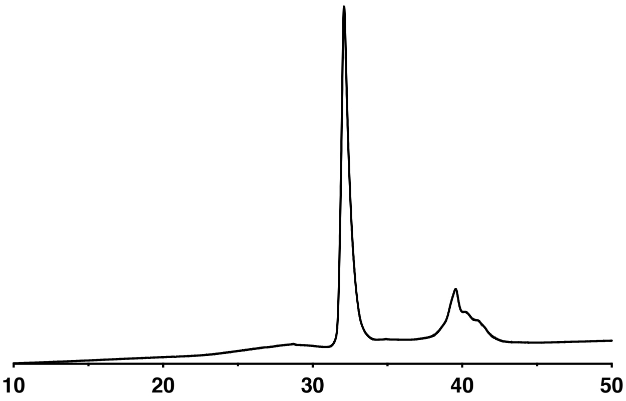

Figure S-1. Crude oxidative folding of TFF3. TFF3 was folded for 48 h in 0.1 M ammonium bicarbonate (NH_4HCO_3) at pH 8.5, 25 ºC forming one predominant isomer.

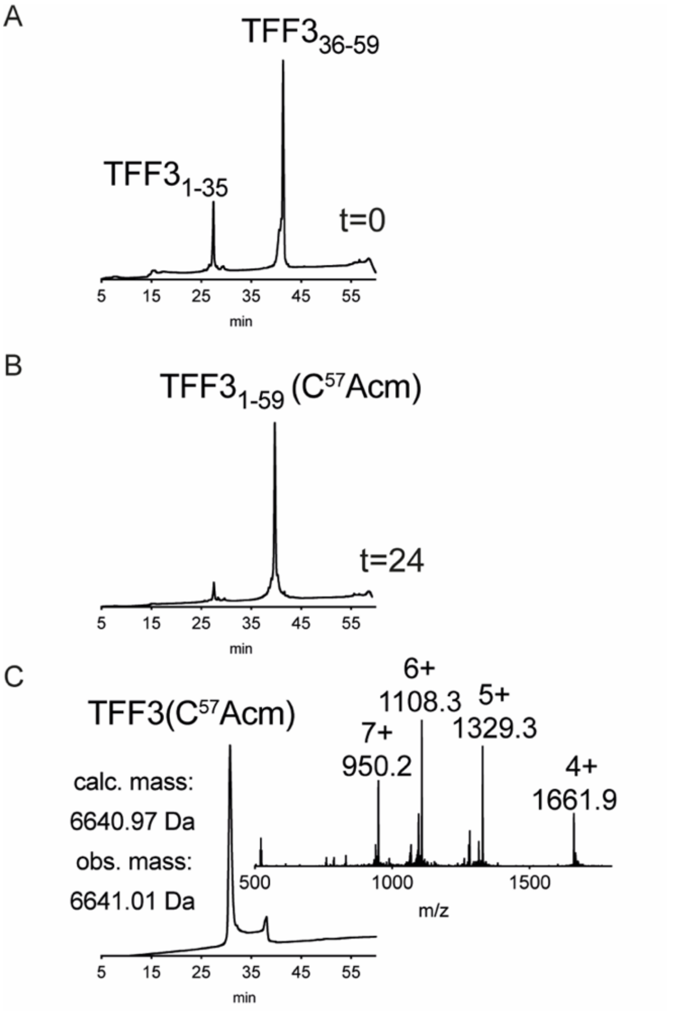

Figure S-2. Chemical synthesis of TFF3(C^{57}Acm). (**A**) Analytical HPLC trace of NCL at 0 h showing the fragments TFF3$_{1-35}$ and TFF3$_{36-59}$(C^{57}Acm). (**B**) Analytical HPLC and MS traces of NCL at 24 h showing the disappearance of the fragments and appearance of full-length reduced TFF3; (**C**) Analytical HPLC and MS traces of folded TFF3(C^{57}Acm) after oxidative folding in 0.1 NH_4HCO_3, pH 8.5 for 48 h.

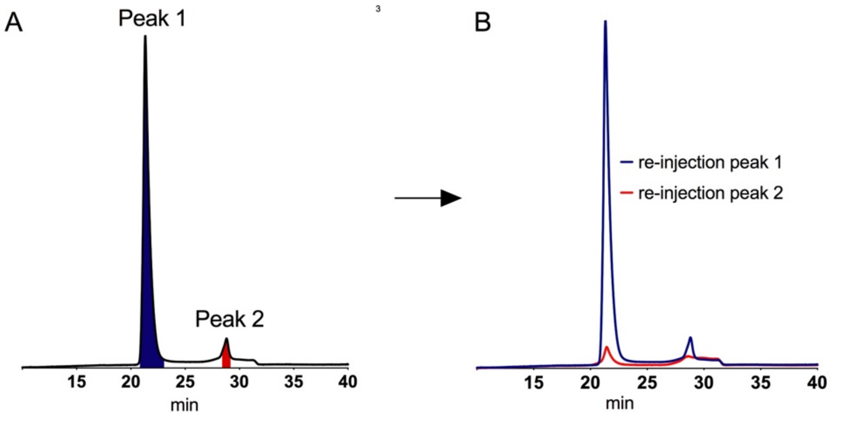

Figure S-3. Analytical HPLC profile of TFF3.(A) TFF3 eluted as two peaks on a C_3-HPLC column. **(B)** The same two-peak profile was observed following the re-injection of each of the peaks independently. Re-injection of peak 1 in blue and re-injection of peak 2 in red.

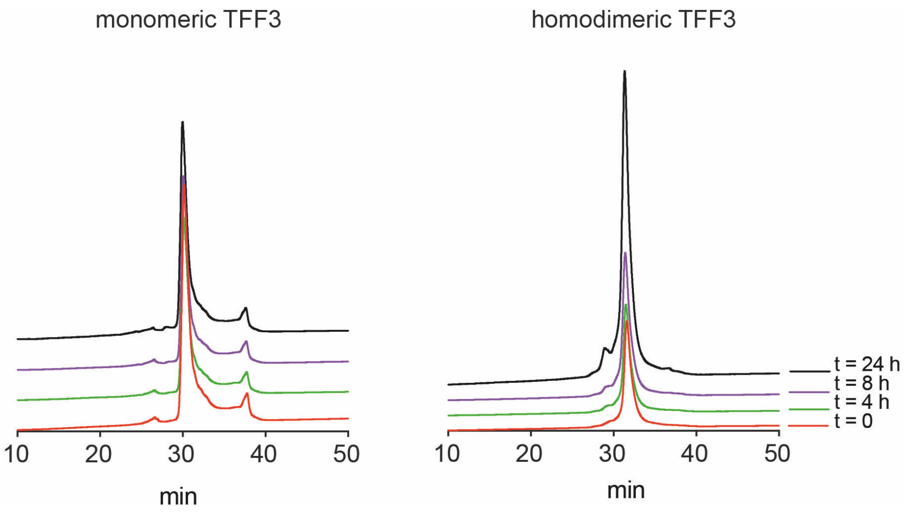

Figure S-4. Stability of TFF3 disulfide bonds in presence of 10 equivalents of reduced glutathione (100 µM) to TFF3 (10 µM) by time-course HPLC.
